# Treatment of Tympanites

**Published:** 1882-03

**Authors:** 


### TREATMENT OF TYMPANITIS.

Tympanitis is a complication of typhoid fever and enteritis, which merits to be treated with care. The late Maurice Reynaud prescribed with great success the following: Nux vomica in powder, six grains; anise-seed in powder, three grains. Mix and divide in two powders, one to be taken in the morning, the other in the evening. M. Reynaud also ordered two tablespoonfuls of powdered charcoal in the course of the day.—*Med. Press and Circular.*

